# Protocol for producing brain-derived neurotrophic factor and neurotrophin-4 in their pro and active form in *Escherichia coli*

**DOI:** 10.1016/j.xpro.2025.103715

**Published:** 2025-03-28

**Authors:** Christoph Holzner, Katharina Böttinger, Christian G. Huber, Elfriede Dall, Hans Brandstetter

**Affiliations:** 1 University of Salzburg, Department of Biosciences and Medical Biology, Hellbrunner Straße 34, 5020 Salzburg, Austria; 2 Center for Tumor Biology and Immunology (CTBI), University of Salzburg, 5020 Salzburg, Austria

**Keywords:** protein biochemistry, protein expression and purification, biotechnology and bioengineering

## Abstract

Brain-derived neurotrophic factor (BDNF) and neurotrophin-4 (NT4) are neurotrophic growth factors, signaling primarily via the tropomyosin receptor kinase B (TrkB). Here, we present a protocol for producing BDNF and NT4 in their pro and active form in *Escherichia coli*. We describe steps for *E. coli* expression, oxidative *in vitro* folding from inclusion bodies, and purification. We then detail procedures for proteolytic activation and characterization of the purified neurotrophins.

For complete details on the use and execution of this protocol, please refer to Holzner et al.^1^

## Highlights

• Instructions for producing proBDNF and proNT4 inclusion bodies in *E. coli*
• Steps for *in vitro* folding of proBDNF and proNT4 with intact disulfide bridges
• Guidance on batch purification and activation of folded proBDNF and proNT4

## Abstract (editor highlights)

Publisher’s note: Undertaking any experimental protocol requires adherence to local institutional guidelines for laboratory safety and ethics.

## Graphical abstract

**Figure:**
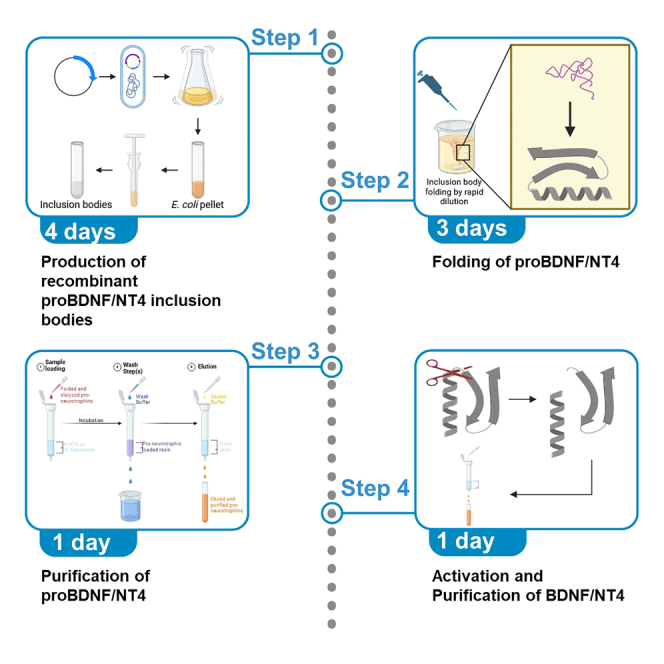

## Before you begin

**Timing: 1–2 days**

This protocol describes the methodology to obtain correctly folded and disulfide-linked neurotrophins proBDNF and proNT4 from E. coli BL21(DE3) Rosetta 2 strain. However, simple BL21 (DE3) *E. coli* work as well. It is important to emphasize that the neurotrophins must be produced in their pro-forms even though the active forms may be required. The pro-peptides prove to fulfill critical chaperone function for proper folding.

1. For one inclusion body pellet prepare 1.8 L of LB-medium (3x 600 mL in 2 L shaker flasks) in advance.
  a. Additionally, prepare at least 1x 50 mL LB-medium in a 250 mL flask for an inoculation culture.
  b. Autoclave both and let them cool down to room temperature (20°C–22°C) before inoculation with *E. coli.*
2. Start an inoculation culture.
  a. Transfer the plasmids into 100 μL chemo competent BL21 (DE3) Rosetta 2 *E coli.*
  b. After the cells recovered in 500 μL LB medium, transfer the cells into the prepared 50 mL LB-medium with selection (100 μg/mL Ampicillin, 20 μg/mL Chloramphenicol).
  c. Grow them over night in a shaker (37°C, 200 rpm).

***Note:*** This is a polyclonal expression system! Monoclonal expression can be set up as well, by plating the recovered cells (in a dilution series) on several LB-agar plates containing 100 μg/mL Ampicillin, 20 μg/mL Chloramphenicol growing them over night. Clearly separated colonies can be picked to inoculate 5 mL LB-medium growing them over night resulting in a monoclonal starting culture.

3. Prepare all buffers described in Materials and Equipment segment in advance and keep/store them at the recommended temperatures.

***Note:*** Buffers that contain urea should always be prepared freshly and kept at 4°C to minimize carbamylation.

4. To measure protein concentrations throughout the whole experiment in a reliable manner it should be done with Bradford Assay. Therefore, prepare a calibration curve with i.e., BSA.

***Note:*** UV/Vis is also viable, but is significantly influenced by the L-Arginine, which is used in the folding buffer in approximately molar concentration and therefore can influence the measured amount of protein. Bradford was found to be more accurate during the refolding experiments with molar concentrations of L-Arginine.

## Key resources table

**Table:** 

REAGENT or RESOURCE	SOURCE	IDENTIFIER
**Antibodies**		

Rabbit polyclonal anti-His 6x (1:10,000)	Abcam	Cat#ab1187

**Bacterial and virus strains**		

XL2-Blue *E. coli*	VWR	Cat#MSPP200150
BL21 (DE3) *E. coli*	Merck Millipore	Cat#69450
BL21 (DE3) Rosetta 2 *E coli*	Merck Millipore	Cat#71400
BL21 (DE3) pLysS *E. coli*	Merck Millipore	Cat#69451

**Chemicals, peptides, and recombinant proteins**		

IPTG	Thermo Fisher Scientific	Cat#15529019
EDTA	Merck Millipore	Cat#324503
GSH	AppliChem	Cat#A2084
GSSG	AppliChem	Cat#A2243
Guanidine hydrochloride	AppliChem	Cat#A1106
L-arginine	Sigma-Aldrich	Cat#A5006
β-mercaptoethanol	Sigma-Aldrich	Cat#M6250
HEPES	AppliChem	Cat#A3724
CaCl2	AppliChem	Cat#A4689
NaCl	AppliChem	Cat#A2942
Sodium phosphate	Sigma-Aldrich	Cat#342483
Na_2_HPO_4_	Merck Millipore	Cat#567550
KH_2_PO_4_	Merck Millipore	Cat#137039
KCl	Merck Millipore	Cat#104936
Bis-Tris	Carl Roth	Cat#9140
MOPS	Sigma-Aldrich	Cat#M1254
Tris base	Sigma-Aldrich	Cat#TRIS-RO
Urea	AppliChem	Cat#A1049
Imidazole	Sigma-Aldrich	Cat#I5513
Triton X-100	AppliChem	Cat#A9778
SP Sepharose	Cytiva	Cat#17072901
Ni-NTA	Thermo Fisher Scientific	Cat#88222
Chloramphenicol	Sigma-Aldrich	Cat#C0378
Ampicillin	Sigma-Aldrich	Cat#A9393
DNase 1	Thermo Fisher Scientific	Cat#EN0525
Xho 1	New England Biolabs	Cat#R0146L
Nde 1	New England Biolabs	Cat# R0111S
Furin	This lab	
Trypsin	Sigma-Aldrich	Cat#T6567

**Critical commercial assays**		

GeneJET plasmid miniprep kit	Thermo Fisher Scientific	Cat#K0502
MinElute gel extraction kit	QIAGEN	Cat#28604

**Deposited data**		

Raw and analyzed data	This paper	

**Recombinant DNA**		

pET22b	Merck Millipore	Cat#69744

**Software and algorithms**		

Xcalibur (v.3.0.63)	Thermo Fisher Scientific	Cat#OPTON-30967
BioPharma Finder (v.1.0 and v.3.0)	Thermo Fisher Scientific	Cat#OPTON-30999
ProSight Lite (v.1.4, build 1.4.6)	Kelleher Research Group	http://prosightlite.northwestern.edu/
Xcalibur Qual Browser (v.4.2.28.14)	Thermo Fisher Scientific	Cat#OPTON-30967

**Other**		

Vivaspin 2, 2,000 MWCO Hydrosart	Sartorius	Cat#VS02H91
Dialysis membrane Nadir/Nalo	Carl Roth	Cat#5104.2
NAP25 DNA purification columns	Cytiva	Cat#17085202
NAP10 DNA purification columns	Cytiva	Cat#17085402

## Materials and equipment

***Note:*** Use molecular biology grade reagents.

**Table:** Digestion Mix

Reagent	Final concentration	Amount in μL
DNA	0.03 μg/μL	20
XhoI	20 U	1
KpnI	20 U	1
Fast Digest Buffer green 10x	1x	4
ddH_2_O	N/A	Fill up to 40
Total	N/A	40

Do not store, always prepare fresh.

**Table:** Ligation Mix

Reagent	Final concentration	Amount in μL
Vector DNA (∼5 kb)	∗	∗
Insert DNA (∼700 bp)	∗	∗
T4 Ligase Buffer 10x	1x	1.5
T4 Ligase	3.5 U	0.7
ddH_2_O	N/A	Fill up to 15 μL
Total	N/A	15

Do not store, always prepare fresh.

∗Determination of the exact amount of vector and insert DNA should be done with a Ligation calculator, like the NEBio Calculator.^2^

**Table:** Wash Buffer 1

Reagent	Final concentration	Amount for 1 L [g]
Tris	50 mM	6.057
NaCl	500 mM	29.220
EDTA	20 mM	5.845

Store at 4°C for 4–8 weeks.

**CRITICAL:** Adjust pH to 8 at 20°C–22°C with HCl or NaOH under a fume hood.

### Wash buffer 2

• Wash Buffer 1 supplemented with: 2% Triton X-100.

**Table:** Solubilization Buffer 1

Reagent	Final concentration	Amount for 1 L [g]
Guanidine	6 M	354.420
Tris	50 mM	6.057
EDTA	20 mM	5.845
β-mercaptoethanol	100 mM	n. A., read below

Store at 20°C–22°C.

**CRITICAL:** Adjust pH to 8.5 at 20°C–22°C with HCl or NaOH under a fume hood.

**CRITICAL:** Do not add β-mercaptoethanol to the buffer! Only add fresh β-mercaptoethanol under a fume hood once the correct volume of Solubilization Buffer 1 is already added to the according inclusion body pellet.

### Solubilization buffer 2

• Solubilization Buffer 1 without β-mercaptoethanol.

**CRITICAL:** Adjust pH to 3.5 at 20°C–22°C with HCl under a fume hood.

**Table:** Dialysis Buffer 1

Reagent	Final concentration	Amount for 2 L [g]
NaCl	50 mM	5.844
EDTA	10 mM	5.845

Store at 4°C for 1 week.

**CRITICAL:** Adjust pH to 4.5 at 20°C–22°C or 4°C with HCl and or NaOH under a fume hood.

**Table:** Folding Buffer

Reagent	Final concentration	Amount for 2 L [g]
L-Arginine	750 mM	261.300
Tris	100 mM	24.228
EDTA	1 mM	0.584
GSH	10 mM	n. A., read below
GSSG	1 mM	n. A., read below

Store at 4°C for 1–2 weeks.

**CRITICAL:** Adjust pH to 9.25 at 4°C under a fume hood.

***Note:*** When dissolving the ingredients, add 50–80 mL of fuming HCl under a fuming hood, to adjust the pH to a window between 9.0 and 9.5. This allows the arginine to dissolve properly and will also cause warming of the mixture. Therefore, first cool the buffer to 4°C, which will increase the pH by ∼0.3 units. Subsequently fine-adjust the pH to 9.25 at 4°C.

**CRITICAL:** Do not add redox agents GSH or GSSG to the buffer! Only add both freshly after the amount of required folding buffer is determined, e.g., 5 mL of solubilized inclusion bodies require 500 mL of fold buffer (1:100 dilution). Calculate how much of the redox agents you have to weigh in for this volume and the specified concentration, add it and dissolve it completely before dripping the solubilized inclusion bodies into the folding buffer.

**Table:** Dialysis Buffer 2

Reagent	Final concentration	Amount for 5 L [g]
HEPES	20 mM	23.830
NaCl	100 mM	29.220

Do not store, always prepare fresh.

**CRITICAL:** Adjust pH to 7.5 at 20°C–22°C with HCl or NaOH under a fume hood.

### Dialysis buffer 3

• Dialysis Buffer 2, but pH is adjusted to pH 7.5 at 4°C!

**Table:** SP-Equilibration Buffer

Reagent	Final concentration	Amount for 100 mL [g]
HEPES	20 mM	0.477
NaCl	50 mM	0.292

Store at 4°C for 2 weeks.

**CRITICAL:** Adjust pH to 7 at 4°C with HCl and/or NaOH under a fume hood.

**Table:** Wash Buffer 3

Reagent	Final concentration	Amount for 1 L [g]
HEPES	20 mM	4.766
NaCl	100 mM	5.844

Store at 4°C for 2 weeks.

**CRITICAL:** Adjust pH to 7.5 at 4°C with HCl and/or NaOH under a fume hood.

**Table:** SP-Elution Buffer

Reagent	Final concentration	Amount for 250 mL [g]
HEPES	20 mM	1.192
NaCl	500 mM	7.305

Store at 4°C for 2 weeks.

**CRITICAL:** Adjust pH to 7.5 at 4°C with HCl and/or NaOH under a fume hood.

**Table:** Ni-Elution Buffer

Reagent	Final concentration	Amount for 250 mL [g]
HEPES	20 mM	1.192
NaCl	100 mM	1.461
Imidazole	400 mM	6.808

Store at 4°C for 2 weeks.

**CRITICAL:** Adjust pH to 7 at 4°C with HCl and/or NaOH under a fume hood.

**Table:** 10x PBS stock solution

Reagent	Final concentration	Amount for 1 L [g]
Na_2_HPO_4_	100 mM	17.799
KH_2_PO_4_	18 mM	2.450
NaCl	1.37 M	80.063
KCl	27 mM	2.013

Store at 20°C–22°C for 4 weeks.

***Note:*** The pH of the 10x stock should be approximately 6.8. When correctly diluted to 1x PBS the pH should change to 7.4.

## Step-by-step method details

***Note:*** This protocol describes the cloning, expression, purification and activation of proBDNF from inclusion bodies to mature BDNF. Additionally, the steps described below also work for proNT4 with a minor change during purification of proNT4. Therefore, unless it is explicitly mentioned, the protocols for (pro)BDNF and (pro)NT4 are interchangeable.

### Production of recombinant proBDNF inclusion bodies

**Timing: 4 days**

This section describes the production of proBDNF inclusion bodies. It includes three major steps: Plasmid preparation (1 day), inclusion body expression (1 day) and inclusion body purification (2 days).

1. Plasmid Preparation.
  ***Note:*** This section describes the preparation of recombinant plasmids containing the proBDNF genes. Plasmid and insert processing follow the general cloning procedures and contain 5 sequential steps: Gene design and synthesis by provider, restriction digestion, gel extraction, ligation and bacterial amplification, and DNA purification.
  a. Order proBDNF and proNT4 genes (ENA database: AAA63483, AAA41728) from a DNA synthesizing company of your choice. Include a 5′ Xho1 and a 3′ Nde1 cleavage site.
    ***Note:*** For this protocol the sequence for proNT4 (#AAA41728) was codon optimized for *E. coli* expression. The FASTA sequence can be found in the supplementary information (Figure S4).
  b. Restriction digestion.
    i. With the help of Xho1 and Nde1 digest the proBDNF inserts from the plasmid they were delivered with and linearize the pET22b vector.
    ii. Set up the reaction in each tube according to the table in the materials and equipment section.
    iii. Incubate the reaction at 37°C for 1 h.
  c. Gel extraction.
    i. Prepare a 0.5% agarose gel with Tris Acetate-EDTA (TAE) buffer.
    ii. Load digestion products into the gel pockets.
    iii. Run the gel at 100 V for 30 min.
    iv. Cut out the desired bands (proBDNF: 699 bp; proNT4: 570 bp; pET22b: ∼5300 bp) from the agarose gel.
    v. Process the gel slices with MinElute Gel Extraction Kit (QIAGEN) according to the manufacturer’s instructions of the gel extraction kit. Other gel extraction kits should work as well.
  d. Ligation.
    i. Calculate the amount of insert needed for 200–300 ng of pET22b in a 3:1 ratio with e.g., the NEBioCalculator.^2^
    ii. Calculate the volumes for pET22b vector and proBDNF insert.
    iii. Pipet these volumes into an appropriate tube.
    iv. Add 1.5 μL T4 Ligase Buffer.
    v. Add according volumes of ddH_2_O to fill up to 14.5 μL.
    vi. Add 0.5 μL of T4 Ligase.
    vii. Incubate at room temperature (20°C–22°C) for 2 h.
  e. Bacterial Amplification in XL2-Blue *E. Coli* and DNA purification.
    i. Transfer ligation product into XL2-Blue *E. Coli* by electroporation.
    ii. Allow the transformed cells to recover in 500 μL pre-warmed (37°C) LB-Medium on a shaker incubator for 1 h.
    iii. Transfer 20 μL of regenerated cells into 4 mL LB-Medium supplemented with 100 μg/mL Ampicillin.
    iv. Grow at 37°C and 200 rpm overnight in a shaker incubator.
    v. Extract plasmid DNA with GeneJet MiniPrep Kit (Thermo Fisher Scientific) according to manufacturer. Other plasmid DNA extraction kits should work as well.
    vi. Send extracted plasmids to an external sequencing company of your choice for confirmation of the correct sequence.
2. Inclusion Body Expression.
  ***Note:*** This section describes the expression of proBDNF inclusion bodies in bacterial cultures and the follow up purification of the inclusion bodies. It contains 3 sequential steps: Start of inoculation culture, expression, and harvest.
  a. Inoculation culture.
    ***Note:*** BL21 (DE3) Rosetta2 E. coli offered the best expression results. However, standard BL21 (DE3) E. coli were able to express proBDNF and proNT4 inclusion bodies as well (figure S1).
    i. Plasmids with sequencing confirmed proBDNF gene are heat shock transferred into BL21 (DE3) Rosetta2 *E. coli.* After regeneration transfer the bacterial cells into 50 mL LB-medium (containing 100 μg/mL Ampicillin and 20 μg/mL Chloramphenicol).
    ii. Grow the bacterial cells in a 37°C incubator over night at 200 rpm shaking.
  b. Expression culture and expression start.
    ***Note:*** 3 X 600 mL of expression culture must be started to gain enough inclusion bodies for one single pellet of inclusion bodies.
    i. Inoculate 600 mL LB-medium (contains 100 μg/mL Ampicillin) in a 2 L shaker flask with 3–4 mL of overnight bacterial inoculation culture.
    ii. Grow in 37°C incubator at 200 rpm shaking to an OD600 of around 0.8–1.
    iii. Induce expression by adding IPTG to a final concentration of 1 mM.
    iv. Induce the protein production of under continuous shaking and 37°C for 3–4 h. The protein was found exclusively in inclusion bodies.
  c. Harvest.
    i. Harvest each 600 mL culture by centrifugation at 4500 x g for 10 min at 4°C in a fixed angle rotor with 1000 mL tubes.
    ii. Discard medium and transfer pellet by scraping it from the bottom of the 1000 mL tube with a spatula into a 50 mL tube.
    iii. Pool 3 pellets into one 50 mL tube.
    iv. Store the pooled pellets at −20°C.
3. Inclusion Body Purification.
  ***Note:*** This section describes the purification of the proBDNF inclusion bodies from the harvested bacterial pellets. It contains 4 sequential steps: Pre-wash, Sonication, Wash and Dialysis.
  a. Pre-Wash.
    i. Thaw pellet and resuspend in 20 mL Wash Buffer 1 by vortexing.
    ii. Centrifuge at 6,000 x g for 10 min at 4°C and discard supernatant.
  b. Sonication.
    i. Resuspend pellet in 30 mL Wash Buffer 1 by vortexing.
    ii. Sonicate at near 100% power, 50% duration, 9 intervals (3 min each) on ice.
    iii. Add DNase (small spatula tip) and MgCl_2_ (final conc. 5 mM).
    iv. Incubate for 30 min at 4°C on roller or shaker incubator.
    v. Add Triton X-100 (final conc. 2%) and shake thoroughly by hand until Triton has dissolved in the tube.
    vi. Centrifuge at 17,500 x g for 15 min at room temperature (20°C–22°C) and discard supernatant.
    vii. Weigh pellet.
      ***Note:*** After the harsh sonication process it is not necessary anymore to keep the sample at 4°C. Therefore, centrifugation and washing at room temperature is possible.
  c. Wash.
    i. Transfer pellet into the Potter homogenizer.
    ii. Add 4 mL per g pellet of Wash Buffer 2 and resuspend the pellet.
    iii. Centrifuge at 17,500 x g for 15 min at 4°C or 20°C–22°C and discard supernatant.
    iv. Repeat step ii and iii.
    v. Change the buffer of step b to Wash Buffer 1 and repeat step ii and iii two more times.
    vi. Weigh pellet after last centrifugation.
  d. Dialysis.
    i. Add 10 mL Solubilization Buffer 1 per g pellet.
    ii. With a magnetic stirrer, stir until pellet is dissolved.
    iii. Add 2 M HCl until ∼ pH3.5 (check with pH strips).
    iv. Centrifuge at 17,500 x g for 30 min at 20°C–22°C.
    v. Transfer supernatant into dialysis membrane (maximum 5 kDa cutoff).
    vi. Dialyze 3 times against 2 L of Dialysis Buffer 1 for a minimum of 6 h each.
    vii. For a quality control the protein must start to precipitate after the second dialysis at the latest and further precipitate at the third dialysis.
    viii. Centrifuge the dialysis product at 17,500 x g for 30 min at 20°C–22°C and discard supernatant.
    ix. Weigh pellet.
    x. Store pellet at −20°C until further use.

### Folding, purification, and activation of proBDNF inclusion bodies

**Timing: 5 days**

This section describes the folding, purification and activation of proBDNF inclusion bodies. It contains 4 major steps: Folding (3 days), Purification (1 day), Activation of proBDNF (4 h) and Purification of mature BDNF (1 day).

4. Folding of proBDNF.
  ***Note:*** This section describes the folding of the proBDNF inclusion bodies into their native and functional structure. It contains four sequential steps: Solubilization, folding by rapid dilution, concentration, and dialysis.
  a. Solubilization.
    i. Solubilize the inclusion body pellet in 4–6 mL per g pellet Solubilization Buffer 2.
    ii. Centrifuge at 17,500 x g for 20 min at 20°C–22°C.
    iii. Transfer supernatant to new 15 mL tube.
    iv. Measure concentration of supernatant with Bradford Assay. The concentration should be at least 10 mg/mL.
      ***Note:*** After solubilizing the pellet, the concentration of inclusion bodies should be in the range of 10 mg/mL and more, which is outside of the linear range of detection for Bradford Assay. For an accurate measurement, dilute test samples of the solubilized inclusion bodies by 1:20 or 1:50 in the Solubilization Buffer 2.
  b. Folding by rapid dilution.
    i. Prepare Folding Buffer 100 times the volume of the solubilized inclusion bodies at 4°C and add a magnetic stirrer.
    ii. Adjust stirring to a slow yet continuous stirring.
    iii. Carefully and slowly drip 50% of the solubilized inclusion body sample into the folding buffer at 4°C while stirring.
    iv. Stir for at least 15 min for complete mixing and incubate for 4 h.
    v. Drip the remaining 50% into the folding buffer at 4°C.
    vi. Stir for at least 15 min for complete mixing and incubate for 4 h.
      ***Note:*** After the second obligatory 4 h incubation step, we recommend incubating the sample overnight for optimal folding.
  c. Concentration and dialysis.
    i. Concentrate the sample to 100–200 mL with a filter system (maximum 5 kDa cutoff) at 4°C. Suitable concentration techniques include preferentially tangential flow filtration^3^ or alternatively concentration in a dialysis tube with MW cutoff of < 5 kDa against high molecular weight PEG (>30 kDa).
    ii. Transfer the concentrate into a fresh dialysis membrane (MW cutoff can be up to 20 kDa, e.g., Nadir/Nalo membrane, Roth, Karlsruhe, Germany).
    iii. Dialyze for 2 h at 20°C–22°C against 5 L Dialysis Buffer 2 under constant stirring.
    iv. Before transferring the dialysis membrane to 4°C and the new buffer, open it at one side and pipet up and down with a 5 mL pipet to mix the sample inside.
    v. Change to 4°C and 5 L Dialysis Buffer 3.
    vi. Continue dialysis overnight.
    vii. Change dialysis buffer again to Dialysis Buffer 2 and repeat step iii.
    viii. Dialyze for 2 h at 20°C–22°C against 5 L Dialysis Buffer 2.
    ix. Centrifuge the dialyzed protein at 17,500 x g for at least 15 min at 4°C.
    x. Transfer dialyzed protein into a 250 mL Schott-bottle at 4°C or on ice.
      **CRITICAL:** Keep sample at 4°C or on ice from now on, given that the protein is now folded!
      **CRITICAL:** To minimize unwanted modifications such as disulfide shuffling, it is very important to directly continue with the purification of the sample!
      ***Note:*** Although the dialysis buffer of choice is HEPES, because of its buffer range, there are other buffer substances that seem to work equally well. Sodium phosphate, Tris, Bis-Tris and MOPS are also suitable to for dialysis of proBDNF or proNT4 (Figure S2). Just keep in mind, that these buffers can influence further purifications!
5. Purification of folded proBDNF and proNT4.
  ***Note:*** This section describes the purification of the folded proBDNF and proNT4 and includes three sequential steps: Batch purification of proBDNF with SP-sepharose, batch purification of proNT4 with Ni-NTA and NAP column desalting.
  **CRITICAL:** Purification of folded proBDNF must be done using an SP-sepharose because it will bind very tightly to Ni-NTA and can only be removed by stripping the NTA beads with EDTA! By contrast, purification of proNT4 must be done using Ni-NTA Sepharose because proNT4 only binds in very small amounts to the SP sepharose in the buffer conditions after the folding!
  a. Batch purification of proBDNF with SP-sepharose.
    i. Transfer 2.5 mL SP-sepharose beads into a 30 mL gravity flow column.
    ii. Equilibrate the Sepharose with 12.5 mL of SP-Equilibration Buffer.
    iii. Remove the buffer by gravity flow.
    iv. Transfer beads into the flask with the dialyzed protein. Typically, this will be ∼200 mL.
    v. Incubate for at least 30 min at 4°C under constant slow stirring.
    vi. Transfer bead-protein suspension back into the gravity flow column.
    vii. Collect flow through in a suitable flask or tube.
      ***Note:*** Although the target protein should now be bound onto the resin in the column, keep the flow through at 4°C until SDS-PAGE confirms that no target protein is in the flow through.
    viii. Wash the loaded beads twice with 12.5 mL of Wash Buffer 3.
    ix. To elute the protein, incubate the loaded beads with 2.5 mL SP-Elution Buffer on a roller incubator for 10 min at 4°C.
    x. Collect the eluted protein by gravity flow and repeat step (ix) 5 more times, resulting in 6 elution fractions.
    xi. Store elutions on ice or at 4°C until desalting on a 2.5 mL NAP25 column.
    xii. Measure the concentration with Bradford Assay. Concentration should be at ∼0.6 mg/mL.
  b. Batch purification of proNT4 with Ni-NTA.
    i. Transfer 2.5 mL Ni-NTA into a 30 mL gravity flow column.
    ii. Equilibrate the Ni-NTA with 12.5 mL of Wash Buffer 3.
    iii. Remove the buffer by gravity flow.
    iv. Transfer beads into the flask with the dialyzed protein.
    v. Incubate for at least 30 min at 4°C under constant slow stirring.
    vi. Transfer bead-protein suspension back into the gravity flow column.
    vii. Collect the flow through in a 250 mL Schott-bottle.
      ***Note:*** Although the target protein should now be bound onto the resin in the column, keep the flow through at 4°C until SDS-PAGE confirms that no target protein is in the flow through.
    viii. Wash the loaded beads twice with 12.5 mL of Wash Buffer 3.
    ix. To elute the protein, incubate the loaded beads with 2.5 mL Ni-Elution Buffer on a roller incubator for 10 min at 4°C.
    x. Elute the protein by gravity flow and repeat step (ix) 5 more times, resulting in 6 elution fractions.
    xi. Store elutions on ice or at 4°C until desalting on 2.5 mL NAP25 column.
    xii. Measure the concentration with Bradford Assay. Concentration should be at ∼0.8 mg/mL.
  c. NAP column desalting.
    ***Note:*** To minimize waste production the NAP columns can be washed with 15 mL Wash Buffer 3 and re-used for another desalting step.
    i. Equilibrate NAP25 columns with 4 times 5 mL Wash Buffer 3.
    ii. Load 2.5 mL elution on column.
      ***Note:*** For proNT4 only desalt elutions 2 to 6 and consider elution 1 as an extra wash step on the Ni-NTA. Usually, elution 1 does not contain a significant amount of proNT4.
    iii. Let it enter the column.
    iv. Add 3.5 mL Wash Buffer 3 and catch flow through.
    v. Pool the flowthroughs in a 50 mL tube.
    vi. Measure the concentration with Bradford Assay. Concentration should be at ∼0.3 mg/mL.
    vii. Store proBDNF and proNT4 at 4°C over night if processing continues the next day, else store at −20°C.
6. Activation of proBDNF.
  This section describes the activation of the purified proBDNF by Furin (4 h).This step can be similarly applied for proNT4. It contains two sequential steps: Activation of proBDNF using furin and the inactivation of Furin.
  a. Activation of proBDNF.
    i. Add CaCl_2_ (final conc.: 5 mM) to the pooled proBDNF.
    ii. Add Furin in a 1:100 (m(Furin):m(NT)) ratio.
    iii. Incubate for at least 4 h at 20°C–22°C on a roller incubator.
  b. Inactivation of Furin.
    i. Add EDTA (final conc.: 10 mM, pH 7.5) to stop furin activity.
    ii. Centrifuge at 17,500 x g for at least 10 min at 4°C.
      ***Note:*** There are publications indicating that neurotrophin activation by Trypsin^4^ and trypsin-like Plasmin^5^ is possible as well. In our experience, trypsin activates proNT4 and proBDNF with some extra processing, which may be irrelevant for many applications and, therefore, is a viable, cheaper and faster (<1 h) activation alternative to furin activation (Figure S3). However, since trypsin tends to trim the termini, we recommend using furin.
7. Purification of mature BDNF.
  This section describes the purification of mature BDNF separating it from its pro-domain and the activator (furin).
  a. Batch purification of BDNF with SP-sepharose.
    i. Transfer 1 mL SP-sepharose beads into a 15 mL gravity flow column.
    ii. Equilibrate the Sepharose by washing with 5 mL of SP-equilibration Buffer.
    iii. Transfer activated BDNF mix into the column with the outlet closed.
    iv. Incubate the completely closed column on a roller incubator for at least 30 min at 4°C.
    v. After incubation, collect flow through in a 15 mL tube.
      ***Note:*** Although the target protein should now be bound onto the resin in the column, keep the flow through at 4°C until SDS-PAGE confirms that no target protein is in the flow through.
      **CRITICAL:** Increase the concentration of NaCl in the elution buffer to 1 M for the elution of BDNF!
    vi. Wash the loaded beads at least 5 times with 10 mL Wash Buffer 3 each.
    vii. To elute the protein, incubate the loaded beads with 1 mL SP-Elution Buffer on a roller incubator for 10 min at 4°C.
    viii. Elute the protein by gravity flow and repeat step (vii) 5 more times, resulting in 6 elution fractions.
    ix. Store elutions at 4°C until desalting and buffer exchange on 1.0 mL NAP10 column, see below.
    x. Measure the concentration with Bradford Assay. Concentration should be at ∼0.2 mg/mL.
      ***Note:*** To further improve the purification results, you may start the washing protocol by 5 additional washing steps with a wash buffer that contains supplementary 1 M Urea.
8. Buffer exchange via NAP10 column.
  This section describes the buffer exchange after the purification of mature BDNF.
  a. Buffer exchange.
    i. Equilibrate NAP10 columns with 4 times 5 mL 1x PBS pH 7.4.
    ii. Load 1 mL elution on column.
    iii. Let it enter the column.
    iv. Add 1.5 mL 1x PBS pH 7.4 and collect flow through.
    v. Store BDNF at −20°C and NT4 at 4°C.
      **CRITICAL:** Do NOT store NT4 at −20°C! NT4 will precipitate while thawing! NT4 stays stable at 4°C for at least 2 weeks!
      **CRITICAL:** Concentration of proBDNF, proNT4, BDNF and NT4 is often accompanied by severe losses; we specifically recommend using a membrane type like Hydrosart from Sartorius. Concentrating of NT4 is usually not needed. Final typical yields of mature NT4 and BDNF are ∼0.2 mg/mL.
      ***Note:*** We recommend to concentrate mature BDNF for more reproducible concentration measurements.

### *In vitro* characterization of (pro)BDNF

**Timing: 1–2 days**

This section describes the *in vitro* characterization of purified (pro)BDNF and (pro)NT4 by SDS-PAGE and mass spectrometry. It contains two major steps: Analysis of purity by SDS-PAGE (4 h) and analysis of molecular weight by mass spectrometry (1 day).

9. Analysis of purity by SDS-PAGE.
  a. Run an SDS-PAGE.

***Note:*** It is recommended to use 15% polyacrylamide gels in order to have a good resolving power for small proteins (<15 kDa) and middle-sized proteins (<50 kDa). Typically, we mix 10 μL samples with 3 μL of 4x SDS loading buffer ± DTT and load these ∼13 μL on the gel. The gel runs for 30–45 min at 120 V just until the loading dye front has left the gel.

***Note:*** Samples do not need to be boiled before loading. Freshly taken samples that are not immediately loaded on a gel can be stored at 4°C. Before the samples are loaded on the gel, bring them to 20°C–22°C and mix by shortly vortexing.

***Note:*** The SDS PAGE samples are particularly helpful for establishing or trouble shooting the protocol. Therefore, we recommend taking samples for SDS-PAGE after expression, folding, all purification and activation steps.

10. Analysis of molecular weight by mass spectrometry.
  a. Mass spectrometry analysis of proBDNF and proNT4.
    i. Use Q-Exactive Plus quadrupole-Orbitrap (Thermo) or similar device coupled with a Vanquish HPLC-system (Thermo) with a Discovery BIO wide pore C18 column (150 × 2.1 mm i.d., 3.0 μm particle size, 300 Å pore size) (Supelco).
    ii. Set mobile phase A to ddH2O + 0.1% formic acid and mobile phase B to acetonitrile+ 0.1% formic acid.
    iii. Prepare 50–100 μg of TCA (Trichloracetic acid)-precipitated proBDNF and proNT4.
    iv. Dissolve in 175 mmol/L ammonium acetate for final concentration of 1 μg/μL.
    v. Inject 3 μg of the dissolved proBDNF/proNT4 sample.
    vi. Run the following gradients: 15.0% B for 3.0 min, 15.0%–90.0% B for 27.0 min, 90.0%–99.0% B for 0.1 min, 99.0% B for 5.0 min, 99.0%–5.0% B for 0.1 min, 5.0% B for 5.0 min.
    vii. Intact proBDNF and proNT4 were sprayed at 3.5–4.5 kV from a HESI-source in positive ion mode. Proteins were analyzed in full MS mode throughout the chromatographic run (0–50 min) over *m/z* range of 1,000–3,500 at a resolution of 140,000 at *m/z* 200.
    viii. Analyze spectra with Xcalibur software.

***Note:*** Full parameters for HPLC and MS can be found in Holzner et al.^1^

## Expected outcomes

Neurotrophins are important factors for understanding neurological functions and investigating neurodegenerative pathologies. Since most neurotrophins are produced using mammalian or insect cell lines, which are costly and time consuming to maintain, we present an E. coli based step-by-step protocol. Following this protocol should yield 300–600 μg of pure, recombinant mature BDNF or NT4 with intact disulfide bridges confirmed by intact mass analysis. As a side product, by using this protocol we also obtain the pro-forms of BDNF and NT4 in a pure form, suitable for further experiments.

**Figure 1:**
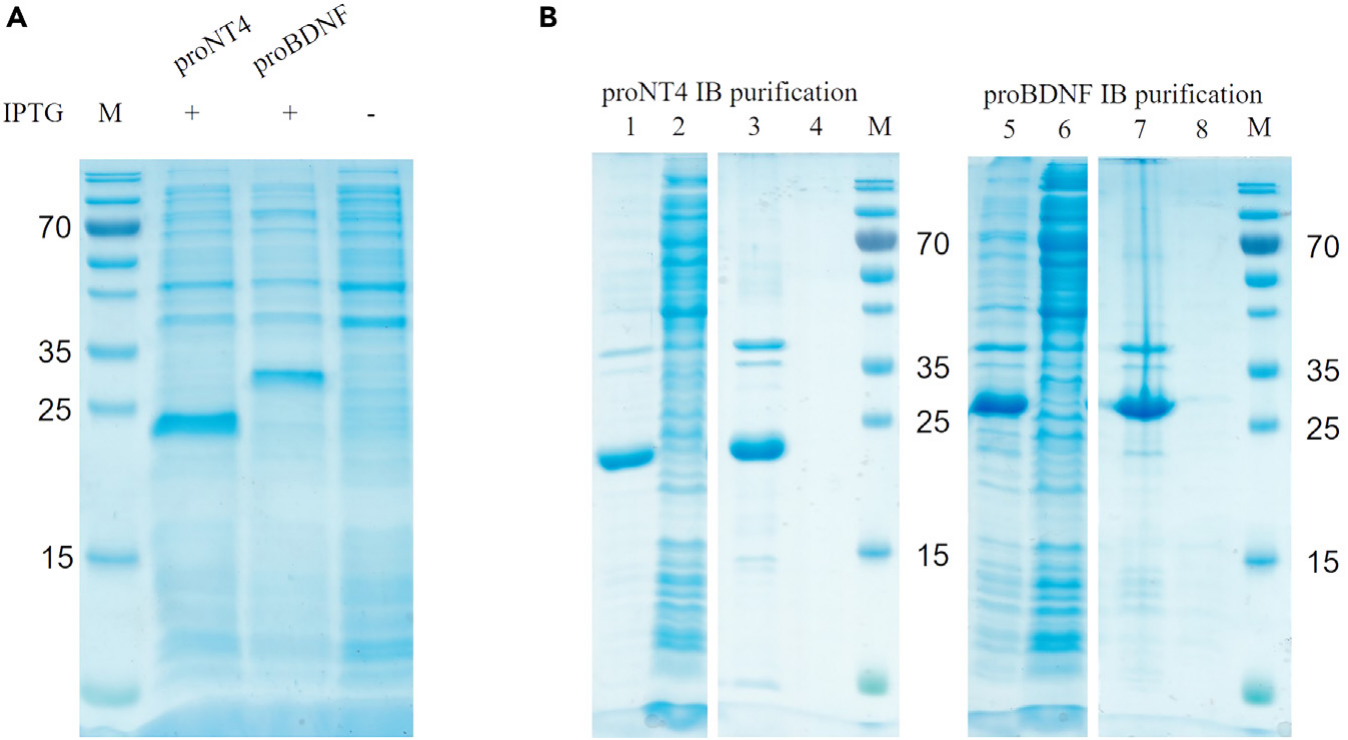
Inclusion Body preparation (A) Expression of pro NT4 and proBDNF. (B) Purification of proBDNF and proNT4. Lane 1, 5: expression pellets; lanes 2, 6: expression supernatants; lanes 3, 7: purified IBs; lanes 4, 8: supernatant after IB purification. M Molecular mass standard (Thermo Fisher Scientific, PageRuler).

Typical experimental intermediate results for important preparation steps:

First, inclusion Body (IB) expression and purification, cf. Figure 1. In Figure 1A, a typical expression of proNT4 (∼25 kDa) and proBDNF inclusion bodies (∼28 kDa) in Rosetta 2 (DE3) *E. coli* is shown. The expression was induced with IPTG and grown for 3 h at 37°C, as described above (step 7). Figure 1B shows the progress of the inclusion body purification of proNT4 and proBDNF.

**Figure 2:**
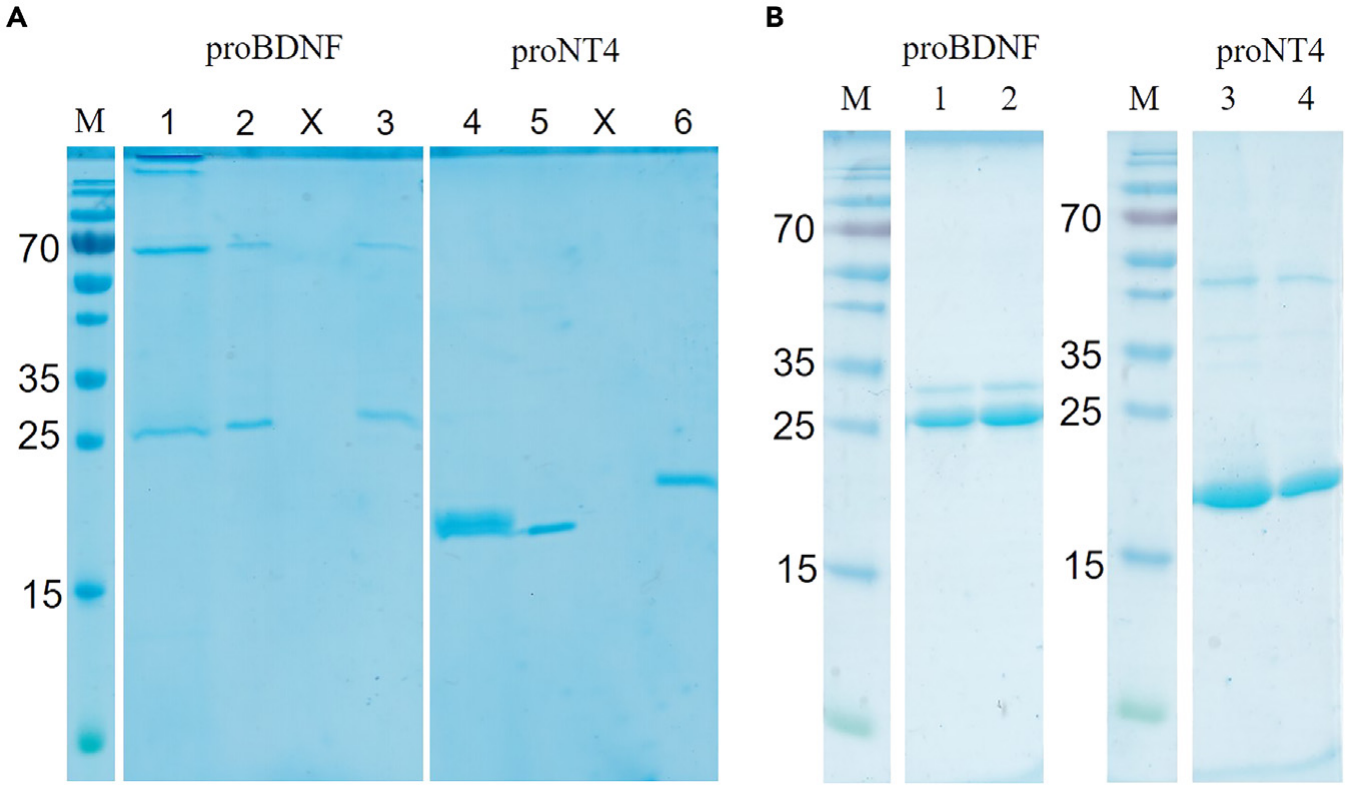
Protein folding and purification (A) M: Molecular mass standard (Thermo Fisher Scientific, PageRuler). Lanes 1, 4: Folded proBDNF and proNT4; Lanes 2, 5: proteins after dialysis; lanes 3, 6: reduced proteins, showing the retarded migration upon disulfide bond reduction. (B) M: Molecular mass standard (Thermo Fisher Scientific, PageRuler). Lanes 1, 3: purified proBDNF and proNT4 after elution from SP-Sepharose and Ni-NTA, respectively; lanes 2, 4: Purified proteins after NAP25 buffer exchange.

Second, folding of inclusion bodies and protein purification, cf. Figure 2. In Figure 2A, non-reducing SDS-PAGEs of proBDNF and proNT4 are shown immediately after the oxidative folding (lane1+4), after dialysis (lane 2 + 5) and compared with the DTT-reduced proBDNF and proNT4 (lane 3 + 6) as a quality control. Initially, a high molecular weight aggregation band is visible for proBDNF, which is removed by precipitation during dialysis, compare lane 1 and 2 as well as 4 and 5. A pronounced migration shift can be observed upon disulfide reduction for proNT4, compare lane 5 and 6, reflecting the more compact structure of the disulfide-bonded proNT4 in lane 5 as compared to the DTT-reduced proNT4 in lane 6. For proBDNF, the effect is less pronounced, but still visible. Figure 2B shows typical elution lanes for proBDNF from SP-Sepharose purification and for proNT4 from Ni-NTA purification, each followed by desalting via a NAP25 column.

**Figure 3:**
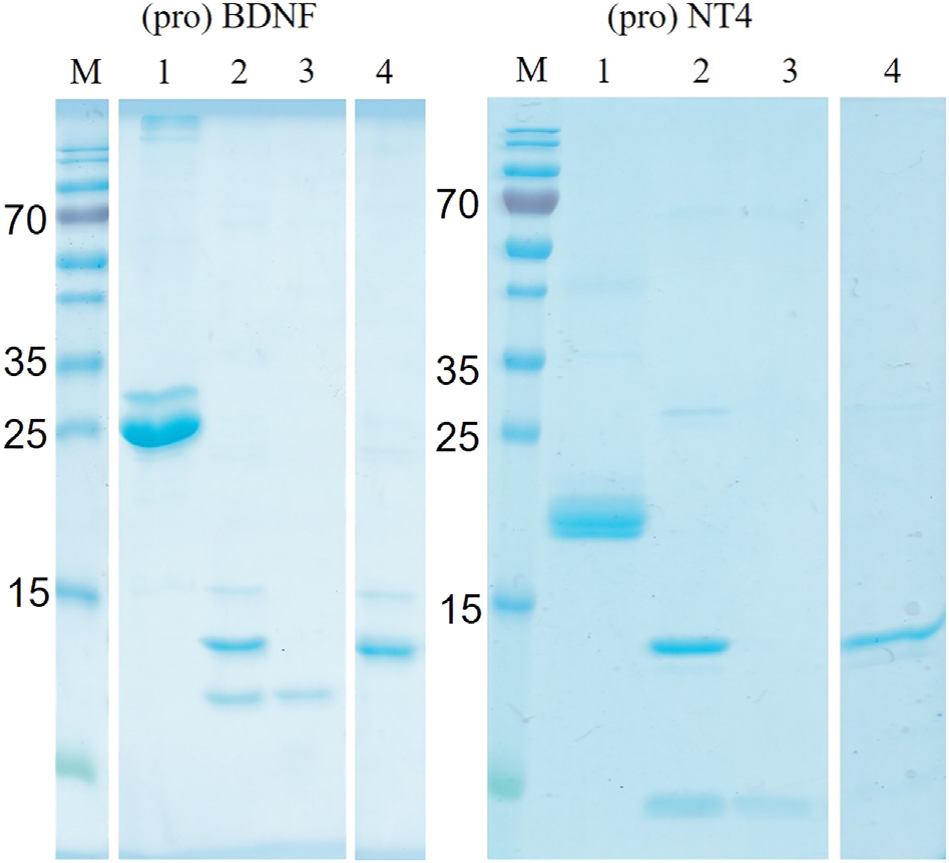
Protein folding and purification M: Molecular mass standard (Thermo Fisher Scientific, PageRuler). Lanes 1, 5: Purified proBDNF and proNT4; lanes 2, 6: furin-activated BDNF and NT4, the released pro-peptides are visible as faint bands below the prominent mature proteins.; lanes 3, 7: Flow-through of the SP-Sepharose purification, separating the pro-peptides; lanes 4, 8: Elutions of the pure mature BDNF and NT4 from the SP-Sepharose.

Third, activation of pro-neurotrophins and purification of the mature forms, cf. Figure 3. Figure 3 shows the furin-catalyzed activation of proBDNF and proNT4, resulting in a characteristic shift on the SDS-PAGE. Additionally, the SP-Sepharose purification of the mature proteins is shown, resulting in apparent homogeneity of either protein.

**Figure 4:**
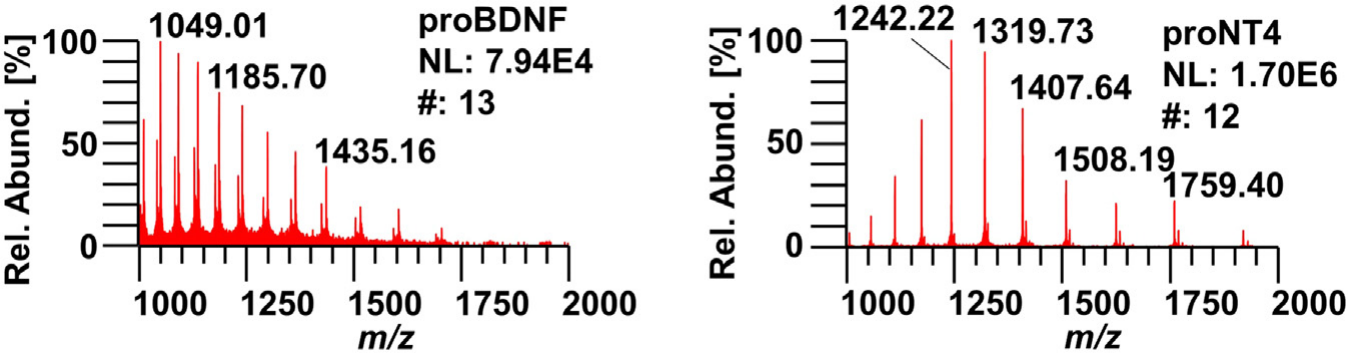
Raw mass spectra of proBDNF and proNT4 Raw mass spectra of proBDNF (left) and proNT4 (right). NL: total ion intensity, normalized to 100%. #: number of scans averaged in the spectrum.

Fourth, quality control of disulfide-linkage by mass spectrometry, cf. Figure 4. Raw mass spectra of proBDNF and proNT4 on a Q-Exactive Plus mass spectrometer are shown. M/z values are clearly resolved, allowing for a unique assignment of intact masses, confirming the expected triple-disulfide bonded proteins, with proBDNF at 27,230.96 Da and pro NT4 at 21,087.58 Da.

## Limitations

As already mentioned in the detailed description part of the protocol, both pro- and mature forms of BDNF and NT4 are adhesive to regenerated cellulose membranes, which may lead to very high losses when concentrating the proteins with membranes of this type. We, therefore, advise to use different types of membranes from different suppliers, for example Hydrosart from Sartorius. With this membrane there are still losses, but concentration can be achieved for proBDNF, proNT4 and BDNF. Concentration losses for NT4 were high also with this membrane type, but feasible, in particular because the protein was highly concentrated (∼0.4 mg/mL) after the purification.

As an extra word of caution, the produced (pro-) neurotrophins BDNF and NT4 were only tested in *in-vitro* experiments like binding affinity measurements.^1^ If cellular or *in vivo* tests are to be performed, we recommend to test the neurotrophin function also by cell assay as described by Rattenholl et al.^4^

## Troubleshooting

### Problem 1

Neurotrophin-DNA insert is not completely digested and pET22b vector is not completely linearized, leading to failed ligation (related to step 1).

### Potential solution

Make sure to incubate the restriction enzymes with the insert and the vector for at least 30 min at 37°C for successful cleavage. For some restriction enzymes the incubation time must be increased to 60 min.

### Problem 2

Expression cultures do not continue to grow after induction of the expression (related to step 2).

### Potential solution

Induction at OD_600_ levels close to 0.8 can be insufficient for getting the cells started with production and continuous growth. If this happens wait for an OD_600_ above 1 before induction.

### Problem 3

Inclusion body pellet after washing does not dissolve in solubilization buffer (related to step 3).

### Potential solution

If the inclusion body pellet after purification from E. coli/washing does not dissolve, make sure that sufficient and fresh β-mercaptoethanol (100 mM) is added. If this does not improve the solubility, increase the temperature of the mixture with a water bath (∼30°C).

### Problem 4

White film-like aggregates forms on surface of folding buffer after first or second 50% of solubilized inclusion bodies are dripped in (related to step 4).

### Potential solution

Remove the film carefully. The film is aggregated inclusion bodies. To avoid aggregation, adjust the stirring speed and slow the speed of dripping in the solubilized inclusion bodies.

### Problem 5

Dialysis after folding and concentrating does not seem to remove arginine (yellowish color remains) and there is no precipitation (related to step 4).

### Potential solution

Increase mass cutoff of your membrane and thoroughly mix the sample inside the membrane before changing to a new dialysis buffer!

### Problem 6

All protein is found in the flow through (related to steps 5, 7).

### Potential solution

Make sure to use fresh or freshly regenerated and correctly stored SP-sepharose or Ni-NTA beads. Also make sure to use SP-sepharose strong cation exchange beads and not Q-sepharose strong anion exchange beads.

## Resource availability

### Lead contact

Further information and requests for resources and reagents should be directed to and will be fulfilled by the lead contact, Hans Brandstetter (johann.brandstetter@plus.ac.at).

### Technical contact

Technical information and requests should be directed to and will be fulfilled by the lead contact, Hans Brandstetter (johann.brandstetter@plus.ac.at) and Christoph Holzner (christoph.holzner@plus.ac.at).

### Materials availability

For this study the gene sequence of proNT4 was codon optimized for E. Coli expression. The new sequence in comparison to the original one can be found in the supplementary information (Figure S4).

### Data and code availability

The data presented in this study are available on request from the first author (Christoph Holzner) and corresponding author (Hans Brandstetter).

## Acknowledgments

This research was funded by the Austrian Science Fund (FWF) (project P26_3186).

We would like to thank Sven O. Dahms for providing the purified furin needed for the activation of the pro-neurotrophins.^6^

## Author contributions

C.H. performed most experiments, analyzed data, and wrote the paper. K.B. carried out MS measurements, interpreted data, and reviewed the manuscript. C.G.H. analyzed data and reviewed the manuscript. E.D. analyzed data and reviewed the manuscript. H.B. supervised the manuscript, analyzed data, and wrote the manuscript.

## Declaration of interests

The authors declare no competing interests.
