# Supplementary material for: Protocol for producing brain-derived neurotrophic factor and neurotrophin-4 in their pro and active form in Escherichia coli
Source: STAR Protoc. 2025 Mar 28;6(2):103715. doi: 10.1016/j.xpro.2025.103715 (PMC11994400; doi:10.1016/j.xpro.2025.103715)
Supplement: Document S1. Figures S1–S4 [file mmc1.pdf]

Supplement Figure 1: Neurotrophin expression tests in different *E. coli* strains, related to step 2.

Although Rosetta 2 (DE3) *E. coli* offer the best expression results for proBDNF and proNT4 inclusion body expression, standard BL21 (DE3) *E. coli* are also viable alternatives and offer a stable expression for 3-5 hours after induction (left side of gel). However, BL21 (DE3) pLysS strains seem to not be able to express inclusion bodies of both neurotrophins (right side of gel).

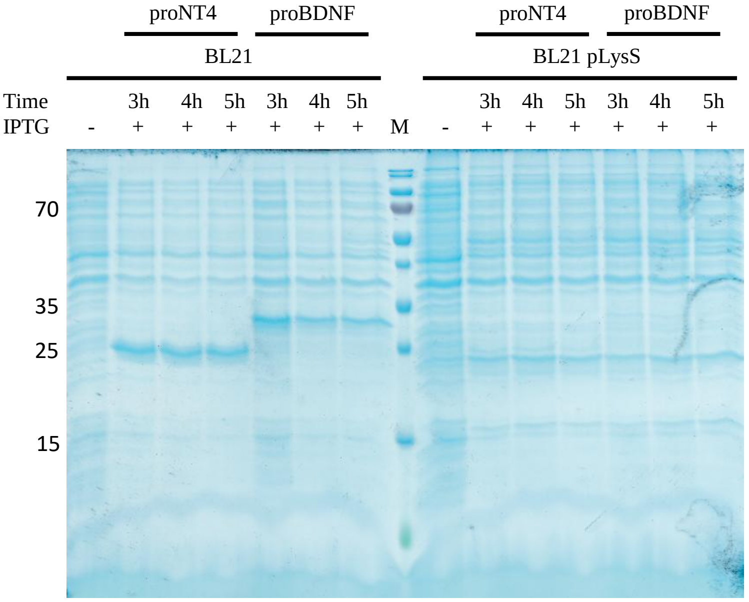

Supplement Figure 2: Buffer condition dialysis tests for proBDNF and proNT4, related to step 4.

Originally proBDNF and proNT4 were dialyzed into a sodium phosphate buffer at pH 7 (1, 7). To test wether there are more suitable buffers the neurotrophins were dialyzed again against sodium phosphate (2, 8), Tris (3, 9), Bis-Tris (4, 10), MOPS (5, 11) and HEPES (6, 12) buffer. High molecular weight bands were best eliminated by dialysis into Bis-Tris, MOPS and HEPES for proBDNF. For proNT4, changing the buffer only showed no to minor improvements compared to the original condition.

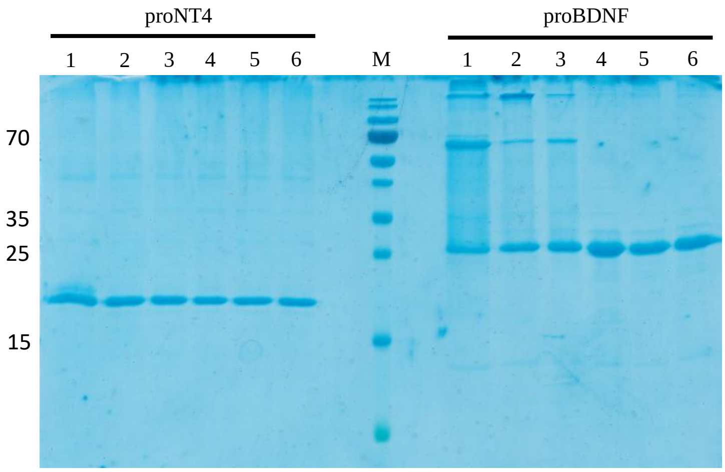

Supplement Figure 3: Activation tests with Furin and Trypsin, related to step 6.

A: Activation tests of proBDNF and proNT4 with Furin. Controls for proNT4 (1) and proBDNF (8) dont contain furin (~70 kDa). Furin is able to successfully process proNT4 and proBDNF within 30 mins (2, 9). Following samples after 1h (3), 4h (4), and 24h (5) show further processing at the C-terminus, cleaving off the 6xHis-tag of mature NT4. Following samples for proBDNF processing (9-12) do not show this further C-terminal cleavage, but the band for the pro-domain of BDNF is clearly visible (~12 kDa). Lanes 6 and 7 show samples of proNT4 and proBDNF processing by Furin after 2h supplemented with DTT before loading on the SDS-PAGE gel.

B: Activation tests for proBDNF and proNT4 with trypsin. Trypsin is able to successfully process proNT4 (1) and proBDNF(4) within 30 mins (3, 5). However, trypsin also seems to cleave off the 6xHis-tag of both mature NT4 and mature BDNF. While proNT4 is detectable in the Immunoblot (1), neither the tag-less trypsin (2) nor the mature NT4 (3) can be detected. Further processing of BDNF after/while activation can be seen when comparing the molecular weight differences with a furin processed sample (6).

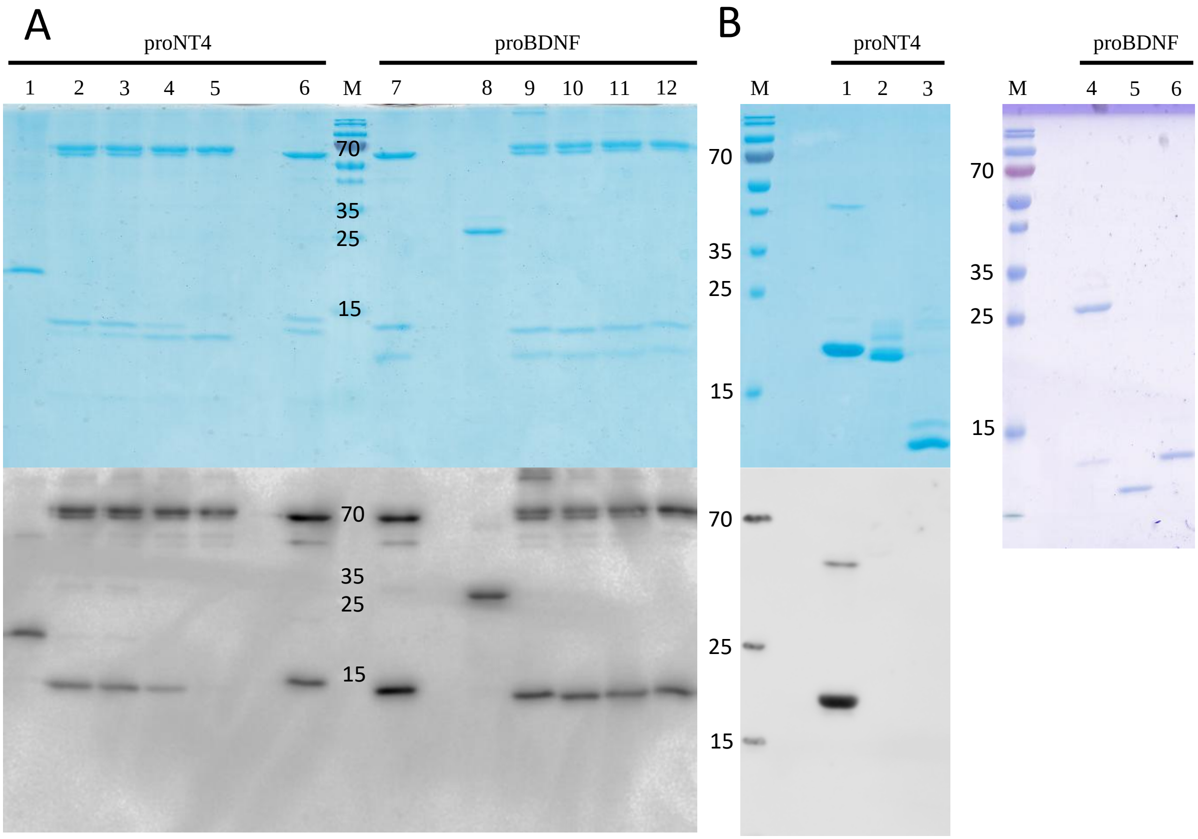

Supplementary Figure 4: ProNT4 gene optimization for E. coli expression, related to step 1

The original proNT4 gene (ENA database: #AAA41728) was manually codon optimized for E. coli expression. The following bases were changed: G66A, C69A, C75A, C78A, C93A, G111A, C129A C165A, C177A, G186A, G204A, C225T, G249A, G276A, T507C, G530C. The changes are indicated by a # in the alignment picture.

```
Original    61   ATGGAGCCCCAACCCCCCTCCTCCACCCTGCCCCCTTTTCTGGCTCCTGAGTGGGACCTC  120
                 |||||#||#|||||#||#||||||||||||||#|||||||||||||||||#|||||||||
Optimized   10   ATGGAACCACAACCACCATCCTCCACCCTGCCACCTTTTCTGGCTCCTGAATGGGACCTC  69

Original    121  TTGTCTCCCCGAGTGGCCCTGTCAAGGGGCACCCCTGCCGGGCCCCCTCTCCTCTTCCTG  180
                 ||||||||#|||||||||||||||||||||||||||||||||||#|||||||||||#|||
Optimized   70   TTGTCTCCACGAGTGGCCCTGTCAAGGGGCACCCCTGCCGGGCCACCTCTCCTCTTTCTG  129

Original    181  CTGGAGGCCGGGGCCTATGGGGAGCCGGCAGGGGCCCCAGCCAACCGCAGCCGGCGCGGG  240
                 |||||#|||||||||||||||||#||||||||||||||||||||#|||||||||||||||
Optimized   130  CTGGAAGCCGGGGCCTATGGGGAACCGGCAGGGGCCCCAGCCAATCGCAGCCGGCGCGGG  189

Original    241  GTGAGTGAGACGGCACCGGCAAGCCGCCGGGGTGAGCTGGCAGTGTGCGATGCAGTGAGT  300
                 ||||||||#||||||||||||||||||||||||||#||||||||||||||||||||||||
Optimized   190  GTGAGTGAAACGGCACCGGCAAGCCGCCGGGGTGAACTGGCAGTGTGCGATGCAGTGAGT  249

Original    301  GGCTGGGTGACCGACCGGCGGACAGCTGTGGACTTGCGTGGGCGCGAGGTGGAGGTGCTG  360
                 ||||||||||||||||||||||||||||||||||||||||||||||||||||||||||||
Optimized   250  GGCTGGGTGACCGACCGGCGGACAGCTGTGGACTTGCGTGGGCGCGAGGTGGAGGTGCTG  309

Original    361  GGCGAGGTGCCTGCGGCAGGTGGCAGTCCCCTGCGTCAGTACTTCTTCGAGACGCGCTGC  420
                 ||||||||||||||||||||||||||||||||||||||||||||||||||||||||||||
Optimized   310  GGCGAGGTGCCTGCGGCAGGTGGCAGTCCCCTGCGTCAGTACTTCTTCGAGACGCGCTGC  369

Original    421  AAGGCCGAAAGCGCTGGGGAAGGTGGCCCAGGTGTGGGCGGAGGGGGCTGTCGCGGCGTG  480
                 ||||||||||||||||||||||||||||||||||||||||||||||||||||||||||||
Optimized   370  AAGGCCGAAAGCGCTGGGGAAGGTGGCCCAGGTGTGGGCGGAGGGGGCTGTCGCGGCGTG  429

Original    481  GATCGGAGGCACTGGCTCTCAGAATGTAAGGCTAAACAGTCCTATGTGCGGGCGTTGACT  540
                 ||||||||||||||||||||||||||#||||||||||||||||||||||#||||||||||
Optimized   430  GATCGGAGGCACTGGCTCTCAGAATGCAAGGCTAAACAGTCCTATGTGCCGGCGTTGACT  489

Original    541  GCAGACTCTCAGGGCCGCGTGGGCTGGCGCTGGATTCGGATCGACACCGCTTGCGTCTGC  600
                 ||||||||||||||||||||||||||||||||||||||||||||||||||||||||||||
Optimized   490  GCAGACTCTCAGGGCCGCGTGGGCTGGCGCTGGATTCGGATCGACACCGCTTGCGTCTGC  549
```
